# When nociception monitoring becomes predictive of surgical outcomes : has the future already become real ?

**DOI:** 10.1007/s10877-024-01139-0

**Published:** 2024-03-05

**Authors:** Mathieu Jeanne

**Affiliations:** 1 Inserm, CIC 1403, Lille, F-59000 France; 2 Univ. Lille, EA 7365 - GRITA, Lille, F-59000 France; 3 CHU Lille, Anesthesia and critical care, Lille, F-59000 France

Perfect control of nociception during general anesthesia may one day enable patients to express no “response to surgical stress” during surgery. Despite some progress in the field of nociception monitoring, this future may be some decades away, and the clinical benefits of an absence of response to stress still needs to be outlined, as one can only suppose that it would result in less hemodynamic impairment, organ dysfunction, digestive malfunction, cognitive impairment, acute and chronic post-operative pain… [1].

In such a scenario, the underlying hypothesis made by the inventors of the Nociceptive Response (NR) Index may be defaulted, as their working hypothesis relies on an hemodynamic model for nociceptive response comprising three input variables: heart rate (HR), systolic blood pressure (SBP) and the perfusion index (PI) of the plethysmographic signal. Using retrospective data of 1054 adult patients ASA status I or II under sevoflurane anesthesia undergoing tympanoplasty (*n* = 729), laparoscopic cholecystectomy (*n* = 195) or open gastrectomy (*n* = 130), one minute averaged values of HR, SBP and PI were analysed with one minute steps from 5 to 15 min after start of surgery [2]. Ordinal logistic regression analysis led to establish probability coefficients for each variable, which were used by the authors to propose an original algorithm called “nociceptive response (NR)”, computed as:

$$NR= \frac{2}{1 +{\text{e}}^{-(0.01\text{H}\text{R}+0.02\text{S}\text{B}\text{P}-0.17\text{P}\text{I})}}-1,$$

The authors have tested the NR algorithm against the mathematical models known as Steven’s power law [3, 4] and Gompertz function [5]. It appears from this original methodology that their working hypothesis relies on nociception being a physiological sense similar to audition and touch, as Steven’s power law and Gompertz function have helped psychophysiologists determine discriminatory thresholds for sound and other human senses. The NR Index has been also measured prospectively in two clinical trials showing lower NR Index values during tympanoplasty (low nociception) than during cholecystectomy (medium nociception) [6].

The reasoning goes as follow: surgical stress, mainly nociception, induces sympathetic activation and a chemical cascade of inflammatory mediators whose influence could be detected in order to anticipate negative outcomes, e.g. after lung resection [7], or gastrointestinal surgery [8], all documented on retrospective massive datasets and with averaging of the NR index over the whole duration of surgery. It is to note that despite its name (“Nociception Response index”), there has been no effort to measure the association between NR Index and any kind of standardized nociception or known endocrine response to nociception.

“Obtaining answers” from a massive retrospective dataset takes usually to defining inclusion and exclusion criteria, types of anesthesia (e.g. general with or without tracheal intubation, loco-regional…), types of drugs (halogenated ethers and/or intravenous only, …). Each criteria retrieve a subset of patients and is presented as a flow chart. Statistical results are usually strongly significant but too often of little or no clinical relevance, mainly because there are no specific statistical tools and no adequate “corrections” for the massive numbers involved. The anesthetic “triple-low” case has quite clearly illustrated these difficulties : Sessler et al. [9] analysed a massive retrospective monocentric dataset of 24,120 patients undergoing general anesthesia for non-cardiac surgery and reported a statistically significant association between triple low (low bispectral index, low mean arterial pressure and low minimum alveolar concentration fraction) cumulative time and postoperative mortality. An editorial accompanying the publication commented that a “triple low may simply reflect the patient’s underlying disease”, that if the relation was causal, the physiopathology leading to the all-cause mortality increase was unclear and that future research should be dedicated to reproducing or refuting the findings [10]. Kertai et al. analysed a dataset of similar patients retrospectively (mono-center, 16,263 cases) but found no statistical association between the so-called triple low cumulative time and perioperative or intermediate-term mortality [11]. Sessler et al. interestingly, chose to test in a prospective randomized trial whether providing “triple low alerts” to clinicians during the course of general anesthesia would reduce 90-day mortality [12]. The methodology was chosen in order to directly answer whether there was a benefit to avoiding or aggressively correcting triple low events, in place of prospectively testing whether there may be causality between triple low events and mortality. The results were negative. Despite the “triple low” false alarm, massive retrospective datasets enable to check for quality markers of routine anesthesia practice and yield fast results. Clinicians must only keep in mind that these results remain doubtful until prospectively proven.

Autonomous Nervous System (ANS) monitoring during general anesthesia is still relatively new. Commercially available “nociception monitors” measure various components of the ANS and its reactions to nociceptive events [13]. These monitors provide real time insight into how administered drugs and analgesic techniques affect the patient and how the very surgical strain affects the patient’s ANS. Because of the absence of gold standard for nociceptive measurements, researchers have had to develop indirect methodologies for comparing the performances of so called “nociception monitors”: electrical stimulation used by myorelaxation monitors have been used as reproducible nociception, plasmatic cortisol levels have been used as indicators of surgical stress [14]. Nociception monitoring and analgesic drug administration guidance through open-loop or closed-loop systems is still actively being researched. Interestingly, it has been shown that at a given time during general anesthesia, heart rate and blood pressure have no predictive value for nociceptive events as compared to nociception monitors [15], which makes a stark contrast with the working hypothesis of Hirose et al., for whom heart rate and blood pressure are two out of three signals used for calculating the response to nociception [2]. The NR Index may work because of the way it is computed and averaged over the whole course of surgery and anesthesia: it may not be related to a singular part of the ANS and may be strongly influenced by endocrine activity and inflammatory response, which was actually the “response” that the authors intended to measure when they first assembled it [6].

In the present edition of the Journal of Clinical Monitoring and Computing, Miyamoto et al. present the retrospective analysis of NR index on a massive dataset of 22,061 patients, with subgroup analysis concerning age groups and surgical risk [16]. This retrospective study is one of several undertaken by the authors in order to assess the clinical utility, potential bias and predictive thresholds of the NR index. Anticipating an unfavourable therapeutic course for a patient during surgery may be one of the most important challenges of modern and future medicine [17]. It may be more adequate to categorize NR Index monitoring as a way to personalize the therapeutic course of a patient rather than a real-time nociception monitor because of its long-term approach to hemodynamic related signals during surgery. Anaesthetists worldwide will probably observe with curiosity the various trials that will either validate or invalidate the added value of NR Index monitoring, similarly to the “triple low” symptoms which, even if they proved unfounded, created a positive questioning into qualitative personalized anaesthesia. In the case of NR index, a favourable effect of real time monitoring leading to actions dedicated to lower the NR index during abdominal laparoscopic surgery seems already to have been demonstred in a monocentric randomized controled trial [18].

## Data Availability

No datasets were generated or analysed during the current study.
